# Perforated marginal ulceration in the setting of single anastomosis duodeno-ileal switch (SADI-S) with conversion to Roux-en-Y gastric bypass and literature review

**DOI:** 10.1093/jscr/rjae828

**Published:** 2025-01-04

**Authors:** Vincent Marcucci, Amanda R Camarda, Veysel Embel, Seth Kipnis

**Affiliations:** Department of Surgery, Jersey Shore University Medical Center, Neptune, NJ, United States; School of Medicine, St. George’s University, Grenada; Department of Surgery, Jersey Shore University Medical Center, Neptune, NJ, United States; Department of Surgery, Jersey Shore University Medical Center, Neptune, NJ, United States; School of Medicine, St. George’s University, Grenada

**Keywords:** bariatric surgery, minimally invasive surgery, marginal ulceration

## Abstract

The single anastomosis duodenal-ileal switch (SADI-S) has become a safe alternative to Roux-en-Y gastric bypass (RYGB) in the treatment for morbid obesity. A known complication after bariatric surgery is the development of marginal ulceration. The current literature demonstrates an overwhelmingly low incidence of ulceration in patients who underwent SADI-S. The management and prevention is an ongoing subject of debate with no clear algorithm. The conversation of SADI-S to RYGB has been accomplished; however, this procedure has not been previously reported for marginal ulceration.

## Introduction

Since its introduction in 2007 as a more simplified version of the initial biliopancreatic diversion with duodenal switch (BPD-DS), the single anastomosis duodenoileal bypass with sleeve gastrectomy (SADI-S) was thought to be just as effective as BPD-DS with potentially less significant postoperative complications. Over the years, the SADI-S procedure has been refined in order to decrease incidence of complications, including nutritional deficiencies, gastric motility issues, and marginal ulcer formation. Reported rates of marginal ulcer formation following bariatric surgery range from 0.6% and 25% with an estimated mean occurrence of 4.6% [1]. The pathophysiology of marginal ulcer formation is multifactorial, however commonly reported risk factors across several bariatric studies include *Helicobacter pylori* infection, tobacco and/or alcohol use, diabetes mellitus, gastric acid hypersecretion, excessive use of non-steroidal anti-inflammatory drugs (NSAIDs), and corticosteroid use [2]. In the acute setting, patients with underlying marginal ulcer may present with fever, tachycardia, and an acute abdomen secondary to gastric perforation [2].

## Case presentation

**Figure 1:**
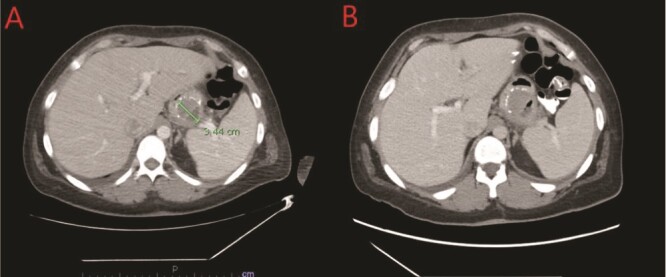
(A) CT showing a complex fluid collection at the proximal greater curvature of the stomach consistent with intramural abscess. (B) Interval improvement in complex fluid collection after endoscopic stent removal.

We report a case of a 39-year-old female, with a past medical history significant for morbid obesity status-post SADI-S ⁓3 years prior at an out-of-state medical center who was transferred to our institution with computed tomography (CT) evidence of an intramural abscess near the proximal greater curvature of the stomach as displayed in Fig. 1A. Our patient had a reported weight loss of 99.8 kg since her bariatric surgery and did not encounter any significant issues until this current episode of symptoms began. She was previously treated with a 2-week course of intravenous (IV) antibiotics with minimal symptomatic improvement prior to transfer to our facility. Notably, the patient was treated with oral corticosteroids ⁓1 month before she initially presented to the hospital for a streptococcal infection. She reported being a non-smoker (neither currently nor previously) and also denied recent use of NSAIDs or proton pump inhibitors (PPIs).

**Figure 2:**
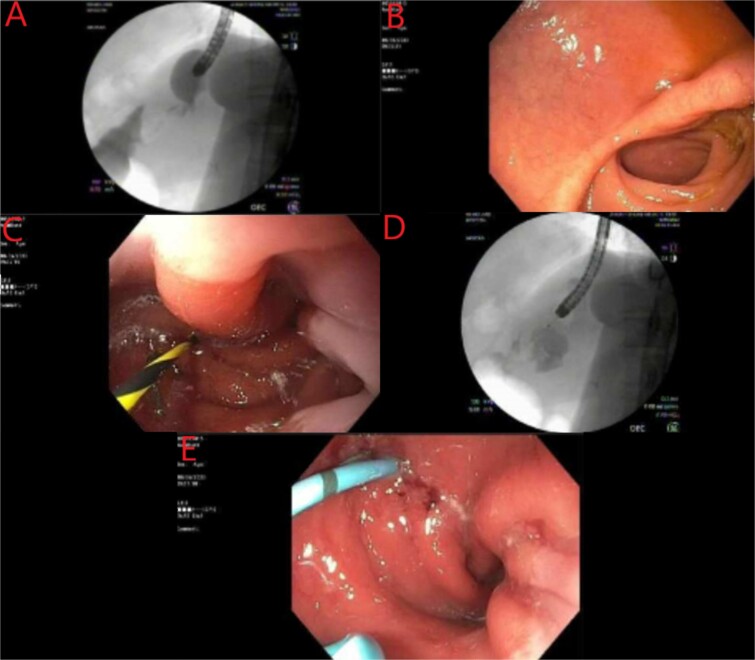
Endoscopic evaluation and stent placement into perforation of the gastric cardia. (A) Delayed imaging of UGI series with no extravasation of contrast. (B) Widely patent duodenal switch lumen. (C) Wire being placed into the fistula tract of the gastric cardia. (D) Contrast filling into the identified abscess cavity. (E) Placement of a double lumen stent through the fistula into the abscess cavity.

A multidisciplinary approach was applied for the complexity of this case. She was maintained on meropenem and she was receiving parenteral nutrition upon arrival to our center, as well as gastrointestinal prophylaxis with proton pump inhibitor and sucralfate therapy. The patient did not have a lactic acidosis or leukocytosis. Nutrition laboratory values were drawn, as shown in Table 1. Within 24 h the patient was brought to the endoscopy suite for luminal evaluation via esophagogastroduodenoscopy (EGD) and a transgastric stent was placed into the abscess cavity. The images from this study can be found in Fig. 2.

**Table 1:** Nutrition labs.

**Parameter**	**Value**	**Reference range**
Iron	91 μg/dL	50–170 μg/dL
Total iron binding capacity	264 μg/dL	250–425 μg/dL
Transferrin	**204 mg/dL**	250–380 mg/dL
Prealbumin	**16 mg/dL**	17–34 mg/dL
Vitamin B1	**195 nmol/L**	78–185 nmol/L
Vitamin B9	13.24 ng/mL	>5.38 ng/mL
Vitamin B12	**1461 pg/mL**	211–911 pg/mL

The patient’s postprocedure course was complicated with severe epigastric abdominal pain, resulting in repeat EGD and stent removal seven days after placement. A follow up CT was completed which demonstrated improvement in the complex fluid collection as shown in Fig. 1B. Her diet was advanced and the patient was tolerating a full liquid diet by the date of her discharge.

Due to the failure of endoscopic management, the decision was made to proceed with surgical intervention due to persistent abdominal pain and diet intolerance. The patient underwent successful laparoscopic Roux-en-Y gastric bypass (RYGB) with drainage of the intraabdominal abscess, and omental patch of the perforated gastric ulcer, as well as repair of a 5-cm hiatal hernia. The perforation was unable to be repaired primarily due to the severity of surrounding inflammation. Adequate stomach proximal to the perforation was identified and utilized successfully for the bypass. Her postoperative course was uncomplicated. An upper gastrointestinal (UGI) series confirmed no signs of contrast extravasation and her diet advanced to full liquids with marked improvement in her abdominal discomfort. She was discharged home on postoperative day #7 without parenteral nutrition.

## Discussion

The incidence of marginal ulceration after bariatric surgery, while uncommon, is a known complication. A 2023 multicenter review between 08 and 22 identified the incidence of marginal ulceration in patients who underwent BPD/DS and single anastomosis duodenal-ileal bypass with sleeve gastrectomy (SADI-S). The rate of marginal ulceration ranged from 0.2% to 1.9% in patients who underwent duodenal switch [3]. A correlation was made between prolonged operative times and preoperative NSAID use in the development of margin ulcers in this cohort [3]. The low occurrence in this population has been considered a result of preserving the pylorus. The pylorus is an important factor in neutralizing acid produced from the stomach that empties into the small bowel after this surgical approach [3, 4].

The rate of annual occurrence of peptic ulceration is <0.3%, which was similar in patients who underwent duodenal switch bariatric surgery [3]. The development of marginal ulceration is patients who have undergone RYGB is upwards of 25% [3, 5]. In an observational study over 5 years evaluating 84 patients (SADI n = 42, OAGB n = 42) with a 36 month follow up identified zero postoperative ulcerations in the SADI cohort compared to 2.4% in the OABG patients [6]. Furthermore, a retrospective study including 1328 patients who underwent SADI-S spanning three countries demonstrated an incidence of postoperative marginal ulceration of 0.1% (2/1328) [7]. The overwhelmingly low incidence of ulceration in this patient population shows the efficacy of this procedure. In regards to our patient, there are several hypotheses as to why she developed a marginal ulcer.

There are several known factors that contribute to the development of marginal ulceration is patients who have undergone bariatric surgery. *H. pylori* infection, tobacco use, and diabetes mellitus, NSAID use were significant predictors of ulceration in this patient population [8]. None of these risk factors were associated with our patient. However, with the use of a short course of oral corticosteroids could very well be the inciting reason for perforation. The impact of corticosteroids on wound healing is well-documented in the literature. Prior studies have discussed the higher rates of marginal ulceration in patients taking corticosteroids. A 2015 study by Coblijn *et al.* identified an odds ratio of 4.46 with corticosteroid use and marginal ulceration after bariatric surgery [9]. However, can steroid use be the sole contributing factor or the main factor? It is commonplace for patients who have undergone bariatric surgery to use PPI as prophylaxis against gastric acid hypersecretion. An occurrence of marginal ulceration was seen in 1.2% of patients on daily PPI prophylaxis compared to a 7.3% in the control group. Our patient was not using PPI prophylaxis [10].

Notably, the various common techniques for gastrojejunal anastomosis have been evaluated. The common methods include; linear stapler, circular stapler, and hand-sewn anastomoses. The data indicates a higher incidence of marginal ulceration when circular stapling devices are utilized [10, 11]. In a 2022 study by Schäfer *et al.* found a significantly higher incidence of ulceration in the patients who underwent RYGB where circular stapler technique was used (26.2%) when compared to the linear stapler cohort (11.3%) [11]. Other studies have found high rates of postoperative bleeding and anastomotic stricture when circular stapling devices are used [11]. While it is unknown the surgical technique utilized in our patient’s initial surgery, it is noteworthy to take this into consideration. There has been little research demonstrating conversion of SADI-S to RYGB due to ulceration. However, this procedure has been accomplished for improvement in bile reflux after SADI-S procedure [12].

Interestingly, the majority of studies evaluating postoperative marginal ulceration in this patient population is largely within 36–48 months after the initial surgery. Our patient, having her procedure 3 years prior leads us to raise the concern there may not be a sufficient timeframe in the current literature to capture long-term complications, which could arise later in the postoperative period. Patients’ nutritional status should always be considered, as in our case with a low prealbumin can certainly increase the risk of ulceration; especially in the setting of corticosteroid use, whether short or long term. Patient selection, extensive preoperative counseling, and postoperative follow up are essential to ensure optimal outcomes in such high-risk surgical patients.
